# Feasibility, safety, and adequacy of research biopsies for cancer clinical trials at an academic medical center

**DOI:** 10.1371/journal.pone.0221065

**Published:** 2019-08-12

**Authors:** Kyoungmin Lee, So Jung Lee, Shinkyo Yoon, Baek-Yeol Ryoo, Sang-We Kim, Sang Hyun Choi, Sang Min Lee, Eun Jin Chae, Yangsoon Park, Se-Jin Jang, Soo-Yeon Park, Young-Kwang Yoon, Seong Ho Park, Tae Won Kim

**Affiliations:** 1 Department of Oncology, Asan Medical Center, University of Ulsan College of Medicine, Seoul, Republic of Korea; 2 Department of Radiology, Asan Medical Center, University of Ulsan College of Medicine, Seoul, Republic of Korea; 3 Department of Pathology, Asan Medical Center, University of Ulsan College of Medicine, Seoul, Republic of Korea; 4 Clinical Trial Center, Asan Institute for Life Sciences, Asan Medical Center, Seoul, Republic of Korea; Seoul National University Hospital, Seoul National University College of Medicine, REPUBLIC OF KOREA

## Abstract

**Objective:**

Research biopsies are an essential component of cancer clinical trials for studying drug efficacy and identifying biomarkers. Site-level clinical investigators, however, do not have access to results on the adequacy of research biopsies for histological or molecular assays, because samples are sent to central labs and the test results are seldom reported back to site-level investigators unless requested. We evaluated the feasibility, safety, and adequacy of research biopsies performed at an academic medical center.

**Materials and methods:**

We retrospectively reviewed the data on 122 research biopsy sessions conducted in 99 patients via percutaneous core needle biopsy for 39 clinical trials from January 2017 to February 2018 at a single institute. We asked the sponsors of each clinical trial for the adequacy of the biopsy samples for histological or molecular assays.

**Results:**

The biopsy success rate was 93.4% (113/122), with nine samples categorized as inadequate for obtaining pathologic diagnosis. Post-biopsy complications occurred in 9.8% (12/122) of biopsies, all of which were mild and completely recovered by the day after the biopsy. The sponsors of clinical trials provided feedbacks on the adequacy of 76 biopsy samples, and noted that a total of 8 biopsy samples from 7 patients were inadequate for analysis, resulting in an adequacy rate of 89.5% (68/76): the reasons for inadequacy were insufficient tumor content for immunohistochemistry (n = 3) and low RNA yield for sequencing (n = 5).

**Conclusion:**

Research biopsies performed at an experienced, multidisciplinary center had acceptable safety for patients as well as practicality in terms of obtaining adequate tissue samples for molecular studies.

## Introduction

Diagnosis and staging of cancers are determined by incorporating results from various tests, and biopsy is one of the most important steps for identifying tumor histology and confirming the presence of metastases for staging [1]. Cancer characteristics vary widely among patients with same type of cancer, and advancement in genome analysis techniques have shown that each patient harbors multiple subpopulation of cancer cells due to genetic mutation, environmental factors, and reversible changes in cellular properties [2]. Therefore, multiple or sequential analysis of tumor materials are necessary to gain deeper understanding of tumor evolution during therapy for each patient, and to bolster drug development strategies in precision medicine [3]. Research biopsies thus serve as a crucial foundation for precision medicine and have become a mandatory component in many clinical trials that are aimed at studying drug effects and identifying relevant biomarkers [4].

Advancement in needle design and in image-guidance technology have improved the safety and efficacy of percutaneous needle biopsies [5], and many studies have investigated the feasibility, safety, and significance of research biopsies in the point of view of site-level pathologists [6–8]. However, there is a lack of data on the actual adequacy of biopsy samples, which is whether they had adequate tumor material for undergoing molecular tests in central labs of clinical trials. A common practice is that most research biopsy samples are sent directly to central labs for correlating molecular tests, and the result of those tests are not reported to the site-level investigators until the end of study periods. Therefore, clinical investigators such as medical oncologists, intervention radiologists, and pathologists do not have sufficient information about the actual adequacy of the research biopsies, which is needed in order to refine and improve biopsy procedures at each site. We therefore evaluated the feasibility, safety, and adequacy of research biopsies carried out for cancer clinical trials at an academic medical center.

## Materials and methods

### Study population and data collection

The protocols of this study were approved by the institutional review board of Asan Medical Center and were conducted in accordance with the Declaration of Helsinki and the Guidelines for Good Clinical Practice. We retrospectively reviewed the electronic medical records of consecutive patients who underwent percutaneous core needle biopsy for correlative clinical trials from January 2017 to February 2018 at the Clinical Trial Center (CTC) in Asan Medical Center, Seoul, Korea. We gathered patient information including sex, age at biopsy, histologic diagnosis, disease extent (recurrent or metastatic), and treatment history. Biopsy information including biopsy site (anatomical location, primary tumor versus metastatic lesion), lesion size, type of imaging guidance, gauge of core needle, and biopsy outcomes (tissue acquisition, complications) were analyzed.

The success of biopsy encompassed technical success and histologic success. Technical success was defined as successful insertion of biopsy needle into target lesion, with cells or tissue present in the specimen [9]. The designated pathologist confirmed and documented histologic success, which was defined as acquisition of adequate amount of pathologic tissue for histologic diagnosis (e.g., tumor cell count ≥ 100 or 50 per protocol). The documented complications were categorized into bleeding (hemoptysis, hematoma, and tract bleeding), embolism, pneumothorax or pneumoperitoneum, infection, and others (e.g., pleural effusion or fluid collection) as well as information on their severity (e.g., delayed discharge, requirements of intensive unit care, and death). The adequacy of biopsy samples was reviewed by the sponsors of each clinical trial who filled out official questionnaires inquiring the purpose of biopsy and tissue adequacy: the purpose of biopsy was categorized into five distinct tests (immunohistochemistry (IHC), fluorescence in situ hybridization (FISH), polymerase chain reaction (PCR), Next-generation sequencing (NGS), ribonucleic acid (RNA) sequencing and others), and the biopsy adequacy was defined as acceptable quality and quantity of tissue samples for pre-planned molecular test of each corresponding trial, and was classified as either adequate or insufficient, the latter of which was considered as molecular failure. The sponsors also provided detailed reasons for categorizing each tissue as insufficient.

### Biopsy technique

Every biopsy was performed on an inpatient basis, and aside from the general informed consent for clinical trials, we also obtained specific informed consent for the biopsy explaining its research, scientific rationale, and potential risk prior to any intervention. Attending oncology physicians requested biopsies to interventional radiologists by informing the purpose of biopsy (clinical research), specific target lesion, and the number of biopsy cores or needle size required for each protocol. If the paired biopsy (pre- and post-treatment) were demanded by the protocol, follow-up biopsies targeted the same lesion as the baseline lesion. The type of guidance imaging, number of biopsy passes, and needle sizes were determined by the interventional radiologist after considering patient safety and apparent tissue yield.

Computed tomography (CT)-guided core biopsies were performed for lung tumors; briefly, a standard co-axial technique was carried out by experienced board-certified thoracic radiologists under CT fluoroscopic guidance with a 64-channel multidetector scanner and used a 19-gauge coaxial introducer and a 20-gauge semi-automated, cutting needle (TSK STERICUT; TSK Laboratory, Tochigi, Japan). Ultrasound (US)-guided core biopsies were performed for other organs such as the liver, peritoneal nodules, and muscles by experienced board-certified abdominal radiologists by using an 18-gauge semi-automated, cutting needle and freehand technique. The biopsy needle was directly advanced into the mass without coaxial technique. Both the thoracic and abdominal radiologists had at least more-than-a-year experience with the respective tumor biopsy procedures (at least a hundred cases) in a high-volume center. Onsite pathologic assessment was not available. The radiologists determined whether the tissue sample from each biopsy was technically well-obtained according to the location of the needle tip and the different gross appearance of the obtained tissue materials from that of healthy tissue of the organ that was harboring the tumor.

### Sample preparation

Core biopsy samples were fixed in neutral buffered formalin and transferred to the pathology lab. After processing and paraffin-embedding using routine histology procedures [10], tissue sections were stained with hematoxylin and eosin and the designated pathologist checked the sample adequacy by estimating tumor cellularity within the specimen. Tissue blocks or unstained slides were then taken to the CTC by clinical research coordinators, prepared for shipment, and delivered to central labs of each clinical trial for subsequent molecular or genetic testing. Except for the domestic trials, the central labs in the global studies were located in Japan, Singapore, Australia, France, Germany, Belgium, and USA. The samples were shipped by air, and the delivery times were approximately 1–2 hours (Japan), 6 hours (Singapore), 10 hours (Australia, France, Germany, Belgium), and 12–14 hours (USA). In all cases, sample preparation and delivery were carried out according to the specified protocols.

### Statistical analysis

Descriptive statistics was used to analyze patient demographics, clinico-pathological characteristics, and biopsy features and outcomes. Qualitative or categorical variables are presented as frequency and proportion, and continuous variables are presented as median and range. Interval between diagnosis and biopsy was defined as follows: in patients not receiving chemotherapy prior to the biopsy, the interval was between the date of diagnosis of metastatic or recurred disease and the date of biopsy; for patients receiving chemotherapy, the interval was between the date of diagnosis of the most recent progressive disease and the date of biopsy. All statistical analyses were performed using IBM SPSS Statistics for Windows, Version 21.0 (IBM Corp., Armonk, NY, USA).

## Results

### Patients and biopsy procedures

Between January 2017 and February 2018, a total of 99 patients underwent percutaneous core needle biopsy at the CTC in Asan Medical Center for correlative 39 cancer trials. Among them, 32 studies listed research biopsies as a mandatory component for enrollment, and the biopsies from 23 trials were research biopsies for integral biomarker study. The median age at the time of biopsy was 57 years (range: 33 to 76 years) and more males (n = 72, 72.7%) were included than females (n = 27, 27.3%). Hepatocellular carcinoma (HCC) (n = 31, 31.3%) was the most common malignancy, followed by non-small cell lung cancer (n = 22, 22.2%) and pancreas cancer (n = 20, 20.2%) (Table 1).

**Table 1:** Patient demographics.

	No.
**Total no. of patients**	99
**Sex**	
Male	72 (72.7%)
Female	27 (27.3%)
**Age, yr, median (range)**	57 (33–76)
**Diagnosis**	
HCC	31 (31.3%)
NSCLC	22 (22.2%)
Pancreas cancer	20 (20.2%)
CRC	12 (12.1%)
CCC	4 (4.0%)
Melanoma	2 (2.0%)
AGC	1 (1.0%)
Bladder cancer	1 (1.0%)
Breast cancer	1 (1.0%)
GB cancer	1 (1.0%)
MUO	1 (1.0%)
Ovarian cancer	1 (1.0%)
RCC	1 (1.0%)
Tonsil cancer	1 (1.0%)

No., number; Yr, year; HCC, hepatocellular carcinoma; NSCLC, non-small cell carcinoma; CRC, colorectal cancer; CCC, cholangiocarcinoma; AGC, advanced gastric cancer; GB, gallbladder; MUO, metastasis of unknown origin; RCC, renal cell carcinoma. Data are presented as no. (%) unless otherwise indicated.

There was a total of 122 biopsy sessions performed in the 99 patients: 23 patients underwent two separate biopsy sessions within the study, including re-biopsies after histological failure at initial biopsy (n = 8), post-treatment biopsies for paired biopsy (n = 14), and additional biopsy for participating in another protocol (n = 1). More than 60% of biopsies were carried out in metastatic lesions (n = 77, 63.1%) and the most frequent sites of biopsy were liver (n = 78, 63.9%), followed by lung (n = 39, 32.0%) and muscles (n = 4, 3.3%). Overall, a median of 3 times of biopsy passes were observed per biopsy and a median of 3 cores with an average maximum length of 0.7 cm were obtained (Table 2).

**Table 2:** Biopsy features.

	No.
**Total no. of biopsy sessions**	122
**Time to biopsy, days, median (range)**	29.5 (2–272)
**Line of chemotherapy before biopsy**	
0	24 (19.7%)
1	46 (37.7%)
2	30 (24.6%)
3 ≤	22 (18.0%)
**Site of biopsy**	
**Primary lesion**	45 (36.9%)
Liver	27
Lung	18
**Metastatic lesion**	77 (63.1%)
Liver	51
Lung	21
Abdominal wall	2
Back	1
Gluteus maximus	1
Peritoneal nodule	1
**Target lesion size, cm, median (range)**	3.0 (0.9–14)
**Guidance imaging modality**	
CT^a^	39 (32.0%)
US	83 (68.0%)
**Biopsy needle**	
18G	85 (69.7%)
20G	37 (30.3%)
**Number of biopsy passes, median (range)**	3 (1–8)
**Number of biopsy cores, median (range)**	3 (1–11)
**Maximal length of biopsy core, cm, median (range)**	0.7 (0.01–1.5)

No., number; CT, computed tomography; US, ultrasonography; G, gauge

^a^All CT procedures were carried out for lung biopsy

Data are presented as no. (%) unless otherwise indicated.

### Biopsy failure and complications

Overall, biopsy success was achieved in 113 of 122 biopsies (93.4%). Out of the nine cases of biopsy failure, only one case was of technical failure, which occurred during CT-guided biopsy in a patient with HCC and metastatic lung lesion. The remaining eight cases of biopsy failure were histological failures, in which either tumor tissues were not obtained or tumor content did not reach the criteria of the corresponding protocol. Liver biopsies accounted for five out of the nine total failed biopsies, and four out of the five liver biopsies were cases of HCC.

Among the 122 biopsies, 110 cases reported no significant complications (90.2%); 12 patients experienced post-biopsy complications, which occurred in either cases of lung biopsies (n = 8, 66.7%) or liver biopsies (n = 4, 33.3%). More than half of post-lung biopsy complications were pneumothorax, and the post-liver biopsy complications were bleeding-related (Table 3). All complications were minor and the patients had complete recovery the day after their biopsy, without any delay in discharge, need for intensive care unit treatment, or death. There were no cases of biopsy failure in patients who experienced post-biopsy complications. All eight patients who underwent re-biopsy due to previous biopsy failure did not experience post-biopsy complications, and sufficient tissues were obtained at the second biopsies.

**Table 3:** Complications.

	No.
**Total no. of cases**	12/122 (9.8%)
**Lung**	8/39 (20.5%)
Pneumothorax	5
Hemoptysis	3
**Liver**	4/78 (5.1%)
Subcapsular hematoma	2
Tract bleeding	1
Pleural effusion	1

No., number. No patient had experienced serious complications such as delayed discharge, need for intensive unit care, or death

### Adequacy of biopsy

**Fig 1:**
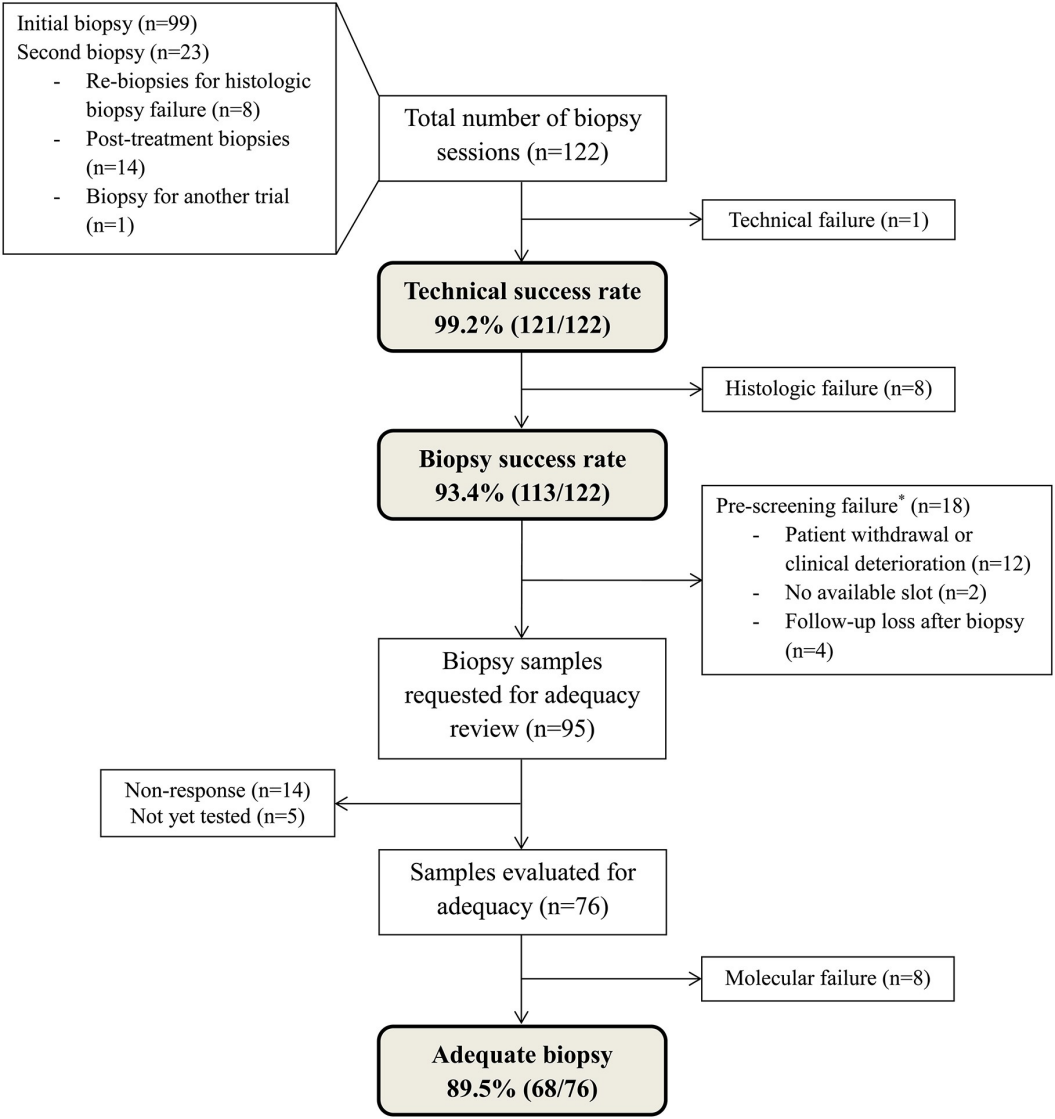
Patient flow diagram. Footnote: *The pre-screening failure cases were samples obtained from patients who met the eligibility criteria, signed an informed consent, underwent biopsy, but failed to start the study treatments.

Among the 113 biopsy success cases, 18 cases of pre-screening failure in whom biopsy was conducted but study treatment was not started were excluded from biopsy adequacy evaluation. Except for the four cases of follow-up loss, most of the pre-screening failures (12/18, 67%) were due to patient withdrawal or clinical deterioration. Also, two patients were not able to start the treatment due to the limited number of available slots. We contacted the sponsors of the clinical trials that incorporated the remaining 95 biopsy samples and asked them to review the adequacy of those samples; after excluding 14 samples that did not receive responses and five samples that had not been tested yet; we gathered adequacy results on 76 biopsy samples (Fig 1).

A total of eight biopsy samples (liver = 4, lung = 4) from seven patients were designated as inadequate by the sponsors, resulting in an adequacy rate of 89.5% (68/76). The reasons for inadequacy included insufficient tumor content (n = 3, 37.5%) and low RNA yield (n = 5, 62.5%) (Table 4). The details of the samples determined as inadequate are shown in S1 Table. Of note, four out of the five samples with low RNA yield were suitable for IHC assays. In cases of 12 paired biopsies conducted at the same sites pre- and post-treatment, five cases (41.7%) were revealed as inadequate for analyzing the changes after treatment (Table 5): three patients had inadequate pre-treatment biopsies results, one had inadequate post-treatment biopsy result, and the other had no adequate biopsy at all.

**Table 4:** Tissue adequacy for pre-planned molecular test.

	Reviewed	Inadequate case	Reason for inadequacy
**Total no. of cases**	76	8 (10.5%)	
**Purpose of biopsy**			
**Single purpose**	54		
IHC	42	3	Insufficient tumor content
PCR	1	0	
NGS	10	0	
RNA sequencing	1	1	Low RNA yield
**Multiple purpose**	22		
IHC	22	0	
FISH	3	0	
PCR	3	0	
NGS	4	0	
RNA sequencing	13	4	Low RNA yield
T-cell receptor sequencing	1	0	
Unknown	7	0	

No., number; IHC, immunohistochemistry; PCR, polymerase chain reaction; NGS, next-generation sequencing; RNA, ribonucleic acid

**Table 5:** Adequacy of pre- and post- research biopsy.

	Pre-treatment biopsy (n = 12)	Post-treatment biopsy (n = 12)	Reason for inadequacy
**Inadequate case**	5/12 (41.7%)		
	4	2	
**Patient No**.			
24	Adequate	Adequate	
25	***Insufficient***	Adequate	Insufficient tumor content
56	***Insufficient***	Adequate	Insufficient tumor content
67	Adequate	Adequate	
69	Adequate	Adequate	
70	Adequate	Adequate	
83	Adequate	Adequate	
88	***Insufficient***	***Insufficient***	Low RNA yield
91	***Insufficient***	Adequate	Low RNA yield
92	Adequate	Adequate	
93	Adequate	Adequate	
94	Adequate	***Insufficient***	Low RNA yield

No., number; RNA, ribonucleic acid

## Discussion

In this single-center study, we found that research biopsies performed percutaneously with image-guidance had 93.4% biopsy success rate, 90.2% complication-free rate, and 89.5% adequacy rate. To the best of our knowledge, this is the first report on the adequacy of research biopsy for pre-planned molecular tests as assessed by the sponsors of clinical trials.

The rate of biopsy success, which was defined as providing sufficient amount of tissue for histopathologic confirmation of malignant lesion, was 93.4% in our study (93.6% in liver biopsies [US-guided], 89.7% in lung biopsies [CT-guided]). This figure is comparable or higher than those of previous studies on liver [11,12] and lung biopsies [13–16]. Various studies have investigated the factors affecting biopsy success rate, and reported that selecting appropriate target lesion is one of the most important steps for a successful biopsy [8,17]. As such, the location, size, and specific radiologic features of each biopsy should be considered simultaneously; therefore, active communication between oncologists and interventional radiologists are helpful for determining the optimal biopsy site [18]. At our medical center, we have designated research biopsy coordinators to facilitate this multidisciplinary communication. These coordinators provide crucial information on the research biopsy to the interventional radiologists, including the purpose, requested method, needle size and minimal number of cores needed for the biopsy. Such facilitated process might have contributed to the high biopsy success rate at our center, as physicians and radiologists were able to share the same viewpoint in selecting the most appropriate target for biopsy. Treatment prior to biopsy also affects biopsy success rate—in the present study, a relatively large number of failures was observed in liver biopsy in HCC patients, most of whom usually received localized treatments such as transarterial chemoembolization, radio-frequency ablation, and radiotherapy, all of which may render tissues more fibrotic and necrotic. Realizing the importance of interdisciplinary discussion on research biopsy success rate, our center holds regular conferences to discuss practical techniques and the optimal practice for image-guided research biopsy.

The molecular adequacy rate of the biopsies (89.5%) from our center is higher than previously reported adequacy rates from other centers. The National Cancer Institute (NCI) [19] and the MD Anderson Cancer Center [17] each reported 74% and 69.9% of adequate rate for NGS genomic testing, respectively. However, such comparison should be interpreted with caution considering the differences in tumor sites and molecular tests among the studies. In our study, obtaining sufficient RNA yield seemed to be the most challenging issue; RNA from formalin-fixed paraffin-embedded (FFPE) tissue suffers from strand breakage and cross-linking during tissue handing and processing; therefore, the length of fixation, type of buffer, and storage time of FFPE blocks are known to impact the RNA sequencing results [20]. In this study, as the sample preparation and delivery were carried out according to the specified protocols of each trials, the storage or shipping time of samples are not likely to have significantly affected the sample qualities. Instead, given that the maximal lengths of all three lung biopsies with low RNA yield were shorter than the median length of the entire biopsy samples, the three lung biopsies may have lacked tumor cells to extract sufficient amount of RNA. In fact, studies have shown that more than 2000 ng of messenger RNA is necessary for NGS [21] and that the minimal tissue requirement value varies according to tissue type [22]. Our results warrant the need for further advancements in RNA extraction technologies.

A common goal of post-treatment biopsies is investigating the mechanism of acquiring resistance to chemotherapy. However, there have been only few reports on the adequacy rates for paired biopsies. In the present study, 14 patients had undergone follow-up biopsies and the adequacy rate of paired biopsies was lower (~60%) than that of total biopsies. Similarly, NCI reported that only ~50% of patients had sufficient paired tissue from paired biopsies [19]. The reason for the lower adequacy of paired biopsies is yet to be clearly defined; a likely possibility is that repeated biopsy of the same site at short intervals may have damaged the cells and tissues, thereby leading to low yield of desired specimens. Thus, studies requiring paired biopsies may need to enroll more patients than usual to ensure appropriate study power, and further prospective evaluations are needed to clearly define the cause of low paired biopsy yields and to resolve that issue.

In our study, post-biopsy complication had occurred in 9.8% of the total cases, which is higher than those of previous studies [6,17]. However, it should be considered that those studies mostly involved biopsies of readily accessible areas such as skin or breast; in contrast, the majority of biopsies conducted in our study were deep tissue biopsies such as lung and liver, which are prone to higher rates of post-biopsy complication. Even so, all complications that occurred in our study population were mild in nature and were completely resolved within a day. Each target organ or site for biopsy carry different risk factors that should be taken into consideration for patient safety; moreover, the disease status and the patients’ underlying conditions would also significantly affect the occurrence of complications. It is expected that the number of early-phase clinical studies involving patients with advanced disease status will continue to increase—nevertheless, there is a notable lack of studies that elucidate the risk factors of biopsy complications in such patients. In this aspect, our study provides useful information on the safety of deep tissue biopsy, which may serve as a basis for future studies evaluating the risk factors of deep tissue biopsy.

Of note, there are ethical concerns regarding research biopsy due to the relative lack of benefits to patients [23]. The importance of molecular biomarker in cancer diagnosis and treatment is more prominent in the era of precision medicine, and biopsy provides the most direct source material to investigate the biomarkers. The majority of research biopsies carried out these days are for integral biomarker studies in which the biopsy result constitutes the eligibility criteria for treatment selection within clinical trials, and thus raise minimal ethical concerns [24,25]. Likewise, 80 biopsies from 23 trials in our current study were obtained for integral biomarker studies. As for the remaining one-thirds of biopsies, which were for correlative science with no immediate benefit to the patients, the patients were fully consulted on the risks and the rationale of the biopsy prior to study registration. Also, the retrospective design of the study as well as its single-center nature has an inherent selection bias; therefore, in order to reduce such bias as much as possible, we investigated all consecutive patients during the study period. Lastly, there was a lack of information on the tumor cellularity within the biopsy samples, which is an important factor together with the core size for calculating the number of cancer cells or expected amount of deoxyribonucleic acid (DNA) or RNA.

Taken together, we demonstrated that research biopsies performed at an experienced, multidisciplinary center had acceptable safety for patients as well as practicality in terms of obtaining adequate tissue samples for molecular studies. Future studies are warranted to assess the factors affecting the quality of research biopsies, and continuous efforts should be made to improve the safety and quality of research biopsies.

## Supporting information

S1 Table Details of the samples determined as inadequate by the sponsors. (DOCX)
