# Supplementary material for: Feasibility, safety, and adequacy of research biopsies for cancer clinical trials at an academic medical center
Source: PLoS One. 2019 Aug 12;14(8):e0221065. doi: 10.1371/journal.pone.0221065 (PMC6690517; doi:10.1371/journal.pone.0221065)
Supplement: S1 Table — (DOCX) [file pone.0221065.s001.docx]

**S1 Table. Details of the samples determined as inadequate by the sponsors**

| Patient no. | **Sex** | **Age** | **Diagnosis** | **Site of biopsy** | **Target lesion size (cm)** | **Guidance imaging modality** | **Biopsy needle (gauge)** | **Number of needle passes** | **Number of biopsy cores** | **Maximal length of biopsy core (cm)** | **Purpose of biopsy** | **Reason for inadequacy** |
| --- | --- | --- | --- | --- | --- | --- | --- | --- | --- | --- | --- | --- |
| 25 | M | 58 | CRC | liver | 4.2 | US | 18 | 4 | 2 | 0.1 | IHC | Not enough tumor content |
| 56 | M | 47 | NSCLC | lung | 1.8 | CT | 20 | 5 | 3 | 0.3 | IHC | Not enough tumor content |
| 57 | F | 42 | CRC | liver | 4.1 | US | 18 | 4 | 3 | 1 | IHC | Not enough tumor content |
| 40 | F | 46 | NSCLC | liver | 4.6 | US | 18 | 2 | 2 | 0.8 | RNA-Seq | Low RNA yield |
| 88 | M | 57 | pancreas cancer | lung | 7 | CT | 20 | 6 | 6 | 0.2 | IHC & RNA-Seq | Low RNA yield |
| 88 | M | 57 | pancreas cancer | lung | 7 | CT | 20 | 4 | 6 | 0.5 | IHC & RNA-Seq | Low RNA yield |
| 91 | M | 52 | pancreas cancer | liver | 2 | US | 18 | 2 | 2 | 0.7 | IHC & RNA-Seq | Low RNA yield |
| 94 | M | 63 | CRC | lung | 2 | CT | 20 | 3 | 9 | 0.5 | IHC & RNA-Seq | Low RNA yield |

No., number; M, male; F, female: CRC, colorectal cancer; NSCLC, non-small cell lung cancer; US, ultrasonography; CT, computed tomography; IHC, immunohistochemistry; RNA-Seq, ribonucleic acid sequencing
